# Forensic Medicine

**Published:** 1837-01

**Authors:** 


### FORENSIC MEDICINE.

*Case of Death from violent Blows.* By Professor Von Siebold, of Göttingen.

[The publication of authentic medico-legal reports has long been a desideratum in our medical literature; and we feel, therefore, that, in occasionally selecting articles of this description for illustration, we shall be acting up to the wishes of a large majority of our readers. It is, besides, in this way only that the practitioner can become acquainted with the importance of Medical Jurisprudence,—a science which, in spite of the obstacles to its cultivation in this country, is making daily and rapid advances. Without entering into a discussion of any of the numerous abstract questions which overload this department of medical knowledge, it is our intention to present to our readers those parts only which have a practical value.]

On the 20th December, 1833, three men of bad character, after a drunken quarrel at an inn, in which the deceased had interfered, left the place on their return home, having persuaded the latter to accompany them. The three men were armed with sticks, while the deceased was defenceless. After they had proceeded a short distance, they suddenly attacked the deceased, beating him severely on the head, body, and legs. Having knocked him down, they dragged him for some distance along the ground, and then renewed their violence. Besides using their sticks, they procured some willow and birch switches, and with these they flogged the deceased, until his dress was torn from his person. According to the depositions, this ill-treatment continued for upwards of half an hour. The deceased then got up, and ran into a thicket, while the prisoners walked away without troubling themselves more about him. Shortly afterwards the deceased was seen by two witnesses, walking along the high road, his dress and person covered with blood. He was compelled to hold up his clothes; and walked in a stooping posture, with a feeble and tottering gait. To their enquiries, he stated that he had been beaten by the prisoners: he then sank exhausted, and in this condition, one of the men having procured a cart, he was driven back to the village whence he had come. Medical aid was immediately called in; but the deceased was then insensible, and in a very short time afterwards he expired.

A medico-legal examination of the body was made on the 22d; and no less than thirty post-mortem appearances are enumerated in the report. The principal of these need only be adverted to, without following the order in which they are given. The skin in the region of the occiput was much ecchymosed and swollen; and beneath was found a considerable extravasation of blood. On the vertex, there was an oblique wound, about one inch and a half long, which penetrated to the bone. Various parts of the face presented marks of bruises and lacerations, but not of a very extensive nature. The upper extremities were covered with marks of contusions from the shoulders to the hands, and there was a corresponding extravasation of blood in the cellular tissue beneath. Similar appearances were met with in the skin covering the glutei and the upper parts of the thighs. On removing the scalp, the bones of the cranium were found uninjured. The vessels of the dura mater and pia mater were much congested; but the brain itself presented no unnatural appearance. The viscera of the chest and abdomen were perfectly healthy. There was an inguinal hernia on the right side, which, on examination, was found to contain omentum and intestine; but these parts were in a healthy condition.

The examiners attributed death to apoplexy, manifested by the congested state of the cerebral vessels, and brought on, in their opinion, partly by external violence and partly by the pain, state of excitement, and fear, under which the deceased was labouring at the time the injuries were received. The ill usage which he had suffered was the sole cause of death: they therefore denounced the prisoners as his murderers. From a want of clearness and precision in this declaration respecting the cause of death, the case was referred to the Medical Faculty of G——, in order that certain queries might be answered. We subjoin these queries, and the answers returned by the faculty:—

1st. *The origin of the injuries found on the body of the deceased, their nature, &c.; taking into consideration the degree of violence and the weapons admitted to have been used by the prisoners?*

The wounds found on the deceased were contused and lacerated; they were situated chiefly about the head and lower parts of the body. The injuries were superficial; there were no fractures of the bones, nor was there any internal morbid change connected with them, with the exception of a congested state of the blood-vessels of the brain. The injuries arose from external violence, and that they were inflicted during life was proved by the extravasations of blood beneath the skin, and the swollen state of the affected parts. The violence used by the prisoners must have been great; and the weapons employed by them sufficiently accounted for the effects observed.

2d. *Was there only one mortal wound, or were there several mortal wounds on the person of the deceased? a. Which were mortal? b. How were they produced?*

In giving an answer to these questions, we must remember that we have to deal with external lacerations and contusions, and one internal morbid change, viz. a congested state of the cerebral vessels. The lacerations and contusions were produced by the direct application of violence to the parts in which they existed; and the cerebral congestion must be regarded as a consequence of the violent contusions on the head. It was here, indeed, that the most serious external injuries existed; for the marks of violence on the extremities and body of the deceased were of a comparatively unimportant nature. Injuries to the head are often followed by the most dangerous results, even where they appear to be small and insignificant. The *force* employed in their infliction must always be taken into consideration, although that force may not produce more than a slight external injury. In the case of the deceased, the violence is proved to have been greater than the mere extent of the injuries about the head would appear to indicate; while the congested state of the cerebral vessels is a proof that the contents of the cranium were severely affected by it. Among the most formidable consequences of blows on the head, especially when inflicted by blunt weapons, as in the case before us, may be mentioned concussion of the brain. This may either destroy life on the spot; produce loss of sense and motion, followed sooner or later by a fatal result; or, under the most favorable circumstances, these symptoms will slowly disappear, and the individual recover. Concussion may prove fatal without leaving any visible changes in the brain; so that when death speedily takes place from violent blows or falls on the head, and, on inspection, no morbid appearances are met with, it is perfectly justifiable to refer death to concussion. In the case of the deceased, however, marks of cerebral disturbance were discovered; there was a general congestion of the cerebral vessels; and this, in our opinion, must be ascribed to the violence used. This congestion must have operated by inducing a state similar to apoplexy, and have destroyed life by fatal compression.

*a.* The injuries to the head generally must be regarded as the cause of death, the external violence being adequate to account for a fatal result; and, although the lacerations and contusions produced were, comparatively speaking, of slight extent, the length of time during which the ill-treatment lasted must be considered as having had a considerable influence in rapidly inducing fatal consequences. While, however, the death of the deceased, is referred to the injuries of the head, the violence inflicted on the back may be admitted to have seriously affected the spinal marrow, and to have assisted in destroying life. No inspection of the spinal column was made; or, had the attention of the examiners been directed to this part, it is not improbable that some such changes as those met with in the coverings of the brain would have been found.

*b.* The answers already given afford a sufficient explanation of the manner in which these wounds on the person of the deceased were produced.

3d. *Of which wound did the deceased die?*

The answer to this question is fully given in the preceding observations. The deceased died from the effects of the violent blows inflicted on his head.

4th. *If no wound of itself was mortal, whether the united effects of the different wounds observed on the deceased were sufficient to account for the destruction of life?*

The solution of this question is in great part involved in that of Question 2. The description of the wounds is sufficient to show that not any one was of itself mortal; but the combination of many blows, and the repetition of the violence upon so important a part of the body as the head, must be looked upon as the cause of a fatal result. Among the blows on the head, those inflicted on the occipital region, from the swelling and extravasation there observed, appear to have been the most serious.

5th. *Is death to be ascribed solely to the ill-treatment experienced by the deceased, or to this, combined with the fact of his having been in the first instance without medical assistance, or to any other accidental circumstances?*

Although it is possible that the life of the deceased might have been preserved, at least for a time, had medical assistance been afforded; yet, for that purpose, a medical practitioner must have been on the spot; and, even then, it is very doubtful whether there would have been any hope of saving his life; since injuries to the head are among the most fatal, and often insidiously destroy life, sooner or later, in spite of the best surgical treatment.—The question may therefore be answered, by asserting that, so far as all medical experience extends, the death of the deceased is to be ascribed solely to the ill-treatment; and that it is in the highest degree improbable, even had medical assistance been instantly afforded him, that he would have recovered. The only accidental circumstance which might perhaps be regarded as having added to the fatal effects of the injuries is the fact of the deceased having been transported some distance in a cart over a rough country-road: but this must be considered as irrelevant to the necessary and general effects of such injuries; the more especially since every attention and care was paid to him after he was discovered, and that, when placed in the cart, the depositions prove that he was already in a hopeless condition.

[This interesting case demands some remarks from us. In the first place, it is clear that the death of the deceased was due to external violence alone; but we do not feel quite satisfied with the manner in which the faculty attempt to explain how the violence operated. In their answer to Quest. 2, they seem to intimate that death was partly due to *concussion* of the brain; but they subsequently declare that the congested state of the cerebral vessels indicated an apoplectic condition, terminating in fatal *compression* of the organ. Now, it does not appear to us that this can be regarded as a case of fatal concussion in the ordinary sense in which that affection of the brain is understood. According to the meaning attached to this term by our first surgical authorities, concussion is indicated by insensibility and derangement of the bodily powers *immediately succeeding the injury.* But the deceased, in the case before us, was able to run away from the prisoners so soon as they left off beating him; and he was afterwards seen walking along the high road. The circumstance of the loss of sense and motion coming on some time after the receipt of the violence, would appear to justify the opinion expressed by the faculty, that death was owing to *compression* of the brain; but the absence of sanguineous effusion, and at the same time the want of the most prominent symptom of compression, *stertor*, which, had it existed, would hardly have been omitted in the report, tend to throw a doubt upon the correctness of this opinion. We are aware that the absence of stertor is not always a proof of there being no compression; since the cases collected by Morgagni show that, even where blood had been effused in considerable quantity, *stertor* was not always present. This, however, is the exception to the rule. (*Cooper's Surgical Dictionary, art. Head.*) It appears to us that in this case, death was referrible to the shock produced on the system through the medium of the brain and nerves, by the continued and repeated violence. This explanation gives us no better idea of the manner in which the violence caused death than that assigned by the faculty; but it has the advantage of avoiding the use of terms which have been long applied to other conditions of the brain.

We learn from this case, what it will be highly important for the medical jurist to bear in mind, in the exercise of his duties; namely, that violence may prove fatal to life without leaving any traces of its effects on the organ primarily affected, (the brain,) and without being indicated by any considerable external marks. It is acknowledged by the faculty, that not a single external wound could be regarded as mortal, or of itself sufficient to account for death; and, therefore, had the body of the deceased been discovered dead, without any history of the ill-treatment to which he had been subjected during life, considerable embarrassment might have existed in the minds of the examiners, especially since the only morbid change observed was congestion of the cerebral vessels. We do not mean to deny that a legitimate inference might have been drawn, even under these circumstances, that the deceased had died from violence to the head; but we believe that it is too much the custom for practitioners in this country to search for some *specially* mortal wound; and, where they do not find such a wound, to abandon the case altogether, as presenting difficulties which medical experience is incapable of surmounting. This sort of prejudice notoriously exists in the minds of our legal authorities, from the coroner to the judge: and thus we find generally, in all investigations relative to murder and manslaughter from wounds, a question is constantly put to the witness, as to which particular wound was mortal. If the witness hesitate to give a positive answer upon this point, the court are apt to acquire a notion that he is not sufficiently informed in his profession. The truth is, a multiplicity of injuries, long continued, although productive of only slight marks externally and internally, are as capable of destroying life as if an individual were at once wounded in a vital organ. This observation particularly applies to wounds of the head; and the case before us is only one of a hundred instances, which might be cited to show that he who sought for some organic lesion essentially mortal to explain death, would be infallibly disappointed. There must always be on these occasions a sort of commendable diffidence in the mind of a practitioner, arising from the obscurity of the subject which he is called upon to investigate; but, let him attend well to the records of experience, and he will feel himself perfectly justified in divesting himself of that which is a mere popular prejudice, and only calculated to mislead him. Our advice then is, that, should such a case as that which we have here presented become a subject of criminal investigation, all circumstantial evidence being wanting, the body should be carefully inspected, in order that it might be seen whether there were any natural causes to account for death; and, in the absence of these, the practitioner should not hesitate to refer death to violence, merely because no single wound would, in a surgical point of view, be pronounced mortal. In pursuing an opposite course, as witnesses are sometimes in the habit of doing at coroner's inquisitions, it must follow that numerous investigations, which might probably lead to the development of crime, will become smothered in their origin.

For further information on the highly important subject of *Wounds*, in a medico-legal sense, we refer to the last four chapters of Mr. Taylor's excellent *Elements of Medical Jurisprudence;* a work which should be in the hands of every practitioner.

*Henke's Zeitschrift für die Staatsarzneikunde. Erlangen,* 1836.

